# 2024: A Year of Nursing Informatics Research in Review

**DOI:** 10.2196/74345

**Published:** 2025-05-07

**Authors:** Elizabeth Borycki

**Affiliations:** 1 School of Health Information Science, University of Victoria, P.O. Box 1700 STN CSC, Victoria, BC, V8W 2Y2, Canada, 1 250-721-8575, 1 250-472-4751

**Keywords:** nursing informatics, health informatics, research, practice, education, trends, artificial intelligence, data science

## Abstract

Each year, nursing informatics researchers contribute to nursing and health informatics knowledge. The year 2024 emerged as yet another year of significant advances. In this editorial, I describe and highlight some of the key trends in nursing informatics research as published in *JMIR Nursing* in 2024. Artificial intelligence (AI), data science, mobile health (mHealth), and the integration of technology into nursing education and practice remain key research themes in the literature. Nursing informatics publications continue to grow in number. A greater number of AI and data science articles are being published, while at the same time, mHealth and technology research continues to be conducted in nursing education and practice contexts.

## Introduction

The year 2024 proved to be a revolutionary year for nursing informatics and *JMIR Nursing*. Nurses, technology practitioners, and researchers who design, develop, and implement technologies used by nurses and nursing informatics specialists are moving forward in the field of study we know as nursing informatics. In 2024, we saw nursing informatics researchers focus on several key areas, namely artificial intelligence (AI), data science, and mobile health (mHealth), as well as integrating technologies into nursing practice and education. In this year’s year-in-review editorial, I describe and highlight some of the key nursing informatics research themes published in *JMIR Nursing* and review 2024’s published articles, using a thematic approach [1]. Findings from the review of the articles revealed many trends that I describe in more detail below.

## Artificial Intelligence

Over the past year, several articles were published that assessed the current state of the science of AI in nursing; the design of AI algorithms; and the effectiveness of AI’s implementation in nursing contexts, such as hospital, community, and long-term care settings. Researchers have investigated the implications of applying AI to lifestyle monitoring in long-term care [2], detecting behavioral disorders [3], identifying depression [4], patient monitoring (eg, movement monitoring for continence care, sleep, and chronic conditions) [5], and supporting nurses’ decision-making [6]. AI-supported technologies, such as robots [7] and chatbots [8], have also been studied and evaluated for their use by nurses in varying care contexts. Other researchers have begun the process of examining AI’s integration into nursing education. Here, there has been an impetus to identify what is important and how to effectively integrate these technologies into nursing education [9].

## Data Science

Data science emerged as a key theme in the nursing informatics research. The development of data models and the analysis of nurse-generated data were considered by researchers as key to supporting nurses’ decision-making in hospital [10], long-term care [11], and community settings [12]. This nursing informatics research aimed to conceptualize and develop electronic health record data models for nurses [13]. Researchers used data-centric approaches to understand and improve nursing workload measures, understand nurses’ and patients’ sentiments regarding COVID-19 [14], collect and present data used in the remote monitoring of patients with COVID-19 [12], and identify patient resources [15]. This research led to new findings that focused on optimizing nursing practice [10-19].

## Ethics and Privacy

Key research areas of concern for nurses who use AI and data science–centric approaches included ethical [19] and privacy considerations [16] associated with using technology. Software testing remained an important aspect of nursing informatics practice to ensure the quality and safety of technologies used in health care [17].

## Nursing Education

Nursing education remained an important theme in the literature [20,21]. The influence of AI on nursing education reflected the need for nurse educators and educational researchers to understand the impacts of these technologies upon nursing education [18]. The role of digital tools and their integration into undergraduate and graduate nursing education were explored [18]. Digital tools used by practicing nurses were studied by researchers [18,19,22]. Here, nurses’ use of multimedia tools to support patient education in cardiac care was a research highlight that emerged [22], and we saw an increase in the number of papers that focused on virtual care in the context of nursing education [20,21].

## mHealth Apps, Tablets, and the Internet

The development of mHealth apps for patients and nurses remained strong [23,24]. Peterson and colleagues [22] studied the effects of a gratitude exercise mindfulness app on neonatal intensive care nurses. Shiyab et al [5] examined nurses’ use of mHealth apps for chronic conditions. Togo et al [25] investigated the effects of mindful breathing using a tablet on nervous system function and sleep. Nurses continued to study mobile apps, software, and devices to determine their influence on patient and nursing outcomes [5,23,24,26,27]. Nurse researchers continued to spearhead mHealth app design and improvements in design, with the aim of improving outcomes. Lastly, nurses evaluated new approaches to finding services on the World Wide Web [15].

In summary, nursing informatics research in 2024 extended our knowledge in the areas of AI and data science. Mobile apps, tablet use, the use of the internet, the integration of nursing informatics into nursing education, and the design of digital tools for nurses and patients continue to be important areas of research.

## Future Research Directions

Nursing informatics research in 2024 advanced in several key areas, including research on the design and use of mobile devices (eg, mHealth tools and tablets) and software apps in the context of nursing practice, education, and administration. In addition to this, several advances in the design and development of data analytics and AI algorithms by nurses have emerged to support and enhance nursing practice. Of importance is the need to study how these technologies can effectively and safely be integrated for use by nurses in acute care, long-term care, and home care settings. Future research will need to focus on how technologies are implemented and incorporated into nurses’ work and patient care, so that there is a strong cognitive-sociotechnical fit between nurse information processing activities, the physical work of nurses, the technologies that are used by nurses, and the patients they care for [1,28,29]. To address these emerging issues in nursing informatics, there is a need to expand funding at the intersection of nursing informatics and AI, data analytics, and mHealth, and research funding extensions need to be made for understanding how these critical new technologies can be added to nursing education and practice [30].

## JMIR Highlights

*JMIR Nursing* also advanced in 2024. *JMIR Nursing* is now indexed in MEDLINE, PubMed, PubMed Central, Directory of Open Access Journals (DOAJ), Scopus, Sherpa Romeo, CINAHL, and the International Academy of Nursing Editors (INANE) directory of nursing journals. More importantly, in 2024, *JMIR Nursing’s* CiteScore rose to 5.2, placing the journal in the 88th percentile. *JMIR Nursing* is now a Q1 journal for general nursing.

## Conclusion

The focus on nursing informatics and emerging technology trends in the field of nursing is proving that technology’s influence upon nursing practice is growing and needs continuous research and support. Nursing informatics researchers and those who study nurses using technology continue to lead the way forward, influencing nursing and health care around the world. Future research directions will need to focus on the integration and incorporation of new and emerging technologies into nursing practice, education, and administration.

## Abbreviations

AI: artificial intelligence
DOAJ: Directory of Open Access Journals
INANE: International Academy of Nursing Editors
mHealth: mobile health
